# Central Ohio Dental Association

**Published:** 1866-01

**Authors:** 


### CENTRAL OHIO DENTAL ASSOCIATION.

We had the pleasure, within a few days, of attending the meeting of this body, at Delaware. There was a very good attendance, though quite a number of the members were absent.

We were really surprised to find the society in such good working order, and so efficient for good in so short a time after its first organization. It is but little over three months since its formation, and this was its third meeting. The members work with a zeal and energy that those of some of our older societies could, with decided advantage, imitate.

They enter upon their work with a will, that shows something must be accomplished. The subjects presented for consideration were practical, and pretty thoroughly discussed. Clinics were given, which were interesting and instructive. Great desire was manifested by the members to aid in any effort calculated to elevate the profession.

Professional elevation seems, just now, to be the "burden of the song," of all our dental associations.

This society will be productive of great good, in that part of the State where its effort shall be put forth, and we predict for it a long and bright career of usefulness.

